# 

**Published:** 1892-12-03

**Authors:** 


					'  "?December 3rd, 1
-a w W ^ WITH EXTRA NURSING SUPPLEMENT.
JW0REN0E.ooo ^ u ^ WITH^?^^r
1 11 Monthly Farts, 6d.; post free, 8d.
Ditto per Annum, 8s., post free.
PRICE TWOPENCE. H pT
*?28, Vol. XIII.?Deo. 3rd, 1892.^ ^ <?> ZJ>
No. 323. Vol. XIII.] Saturday
Decembeb 3rd, 1892.
[Twopenor.

				

